# Cervicogenic-Like Headache as the First Symptom of Acromegaly

**DOI:** 10.7759/cureus.60599

**Published:** 2024-05-19

**Authors:** Adroaldo Rossetti, Carolina Orge, Vitor Melo, Ailton Melo

**Affiliations:** 1 Neurological Surgery, Federal University of Bahia, Salvador, BRA; 2 Speech Therapy, Federal University of Bahia, Salvador, BRA; 3 School of Medicine, Universidade Salvador, Salvador, BRA; 4 Neurology, Federal University of Bahia, Salvador, BRA

**Keywords:** acromegaly symptoms, pituitary tumor, acromegaly, cervicogenic headache, headache

## Abstract

Headache is a frequent symptom in patients with acromegaly; however, it has never been described as a cervicogenic-like headache.

This paper reports on an 18-year-old Brazilian man with a four-year history of unilateral headaches characterized as a sensation of tightness or pressure in the right nuchal region spreading across the forehead. An MRI of the brain revealed a pituitary tumor and a transsphenoidal surgical resection of the macroadenoma was performed. During follow-up, he reported a complete relief of headaches after one week of surgery, persisting for six months. This paper shows a cervicogenic-like headache as the first symptom of acromegaly and the improvement of symptoms after surgery.

## Introduction

Acromegaly is a rare disorder characterized by hypersecretion of the growth hormone (GH) and its peripheral target hormone, insulin-like growth factor (IGF1). Diagnosis of acromegaly is usually made after 3 to 6 years of initial symptoms, and at the time of diagnosis, several comorbidities, such as orofacial changes, osteoarthropathy, diabetes, and cardiovascular diseases, are present [1]. Headache is a frequent symptom in patients with acromegaly, present in up to 70% of the cases. Although sometimes associated with dural meningeal stretch or sellar invasion, the cause of headache is still a matter of controversy [2]. In a previous study, occipital headache was present in 29% of patients with pituitary tumors, and 63% of them had unilateral headaches, which were sidelocked. After surgery, 49% improved their headache [3]. In a systematic review, the authors observed that headache was not correlated with the volume of pituitary tumors, and there was also no association with cavernous sinus invasion [4]. Headache as the first symptom of acromegaly was reported in a 38-year-old woman with a previous diagnosis of rheumatoid arthritis. In this case, the IGF1 level normalized, and the headache improved after surgery [5]. This paper reports the case of an 18-year-old male who had unilateral occipital headache as the first symptom of acromegaly and improved after surgery. We discuss the compression of the C2 and C3 afferent nerves as one possible etiology of headache in patients with acromegaly.

## Case presentation

**Figure 1:**
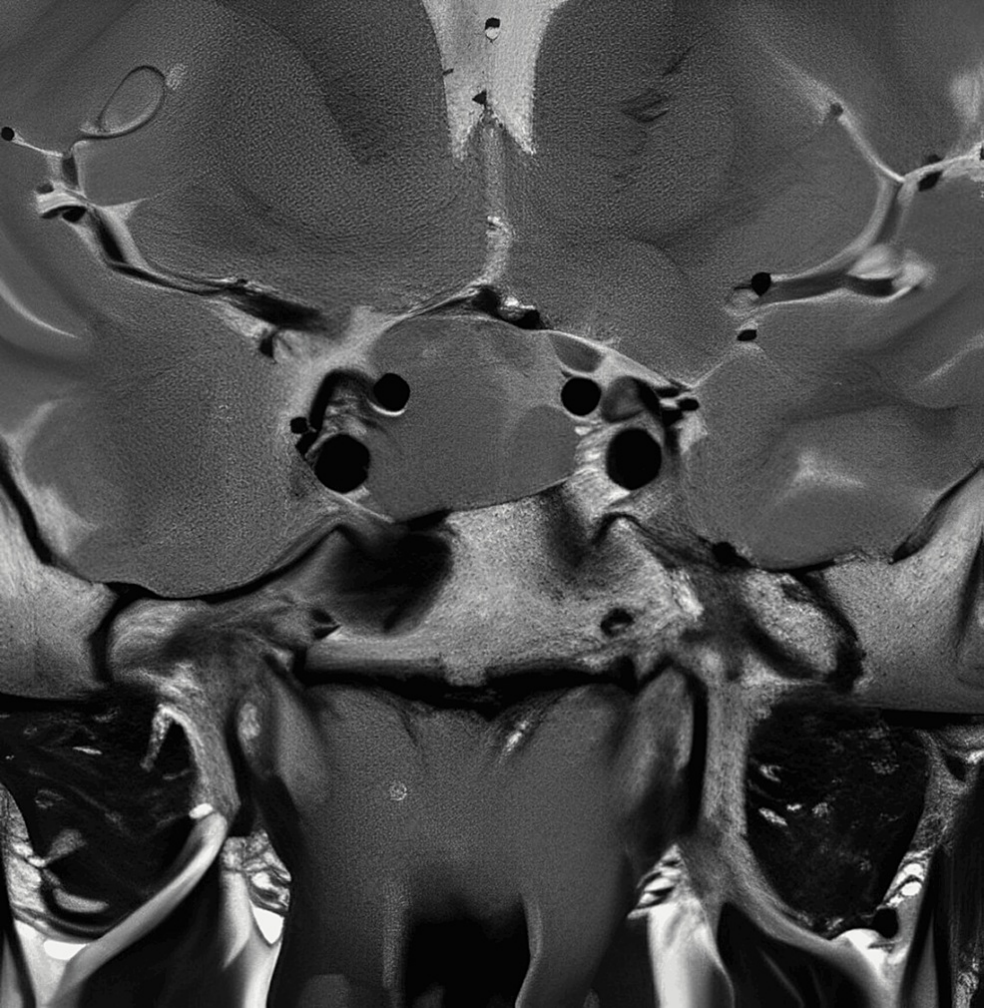
Coronal T2-weighted magnetic resonance image of the brain in an 18-year-old patient showing pituitary macroadenoma.

An 18-year-old male had nuchal pain in the right side of the head for four years without obvious cause. The headache was characterized as a sensation of tightness or pressure in the right nuchal region, spreading across the forehead and worsening with exercises such as soccer or working in agriculture. His pain increased during these years, and once or twice a week he had important episodes of awakening headaches for about 3 to 5 hours, which didn't improve after ordinary painkillers. He never had autonomic manifestations such as miosis, red eyes, or tears. He visited several local primary care clinics with no diagnosis until his mother insisted on performing an image examination. During the clinical visits, no sleep, respiratory, cardiovascular, abdominal, or kidney problems were observed. He also didn't complain of skeletal pain, and there was no bone or joint disease. After four years, he had a brain CT scan that showed a sellar tumor. Subsequent pituitary MRI with contrast showed a sellar tumor measuring 3.0 x 2.3 x 2.9 cm that compressed the pituitary gland with no invasion of the cavernous sinus (Figure 1). He was admitted to the Edgard Santos University Teaching Hospital of the Federal University of Bahia, and the Craniofacial Pain Team was asked to examine him. The neurological examination was normal, including muscle strength. His laboratory exams showed increased levels of GH and IGF1. He underwent endoscopy-assisted transsphenoidal resection of a pituitary adenoma with a satisfactory surgical result. The post-operative pathological diagnosis revealed a pituitary GH adenoma with low prolactin expression. One month after surgery, he reported significant pain relief, even during soccer or hard physical work. He also reported a decrease in body weight and edema. 

## Discussion

Headache is a prevalent symptom in patients with pituitary tumors, mainly in those with acromegaly; however, as far as we are concerned, this is the first case of a patient for whom headache was the symptom responsible for the diagnosis. There are some controversies regarding the cause of headaches in patients with acromegaly, despite some authors speculating sinus invasion, dura matter stretch, and volume of the tumor as probable risk factors [2]. In a large series of patients studied before and after surgery, no correlation was observed between tumor volume and headache, nor was there an association of cranial pain with sellar invasion [4]. Unilateral occipital headache spreading throughout the vertex to temporal and frontal regions and behind the eyes has been described as one of the most common cranial pains in acromegalic patients, similar to what we observed in our case [3]. Sometimes, the headache in these patients is characterized as migraine, hemicrania continua, SUNCT, or cluster headache, probable because of its association with autonomic symptoms [6]. In our opinion, the possibility of a cervicogenic headache must also be considered in some clinical situations, as we observed in the present case, which fulfilled the diagnostic criteria according to the third edition of the International Classification of Headache Disorders (ICHD-3) [7].

Another point to be considered is the fact that some authors have performed lidocaine block of the greater occipital nerve (GON) or lower occipital nerve (LON) with consequent improvement of the headache in patients with acromegaly [6], an approach used to perform the diagnosis of cervicogenic headache [8].

As has been stated before, the increase of GH can modify bone and joint structures in the craniocervical junction of patients with acromegaly [9-10]. Furthermore, this hormone is responsible for water retention and the consequent enlargement of soft tissues such as muscles and ligaments. It is known that afferents to the C2 and C3 roots, such as the GON and LON, can be compressed by the skeletal structures that compose the craniocervical junction, and these nerves can also be entrapped or compressed when they pierce the splenius capitis, obliquos, semispinalis capitis, transverse, or trapezius muscles [11]. In our opinion, as the secretion of GH interferes with hole homeostasis, determining bone growth as well as metabolic disturbances with consequent muscle edema, the swelling of these muscles can determine entrapment of the nerves as it pierces or passes through these structures. The afferent roots of C2 and C3 can also be compressed when passing through the craniocervical joint, as has been described elsewhere [11-12].

In summary, we advocate that one possibility of headache in acromegalic pain results from GON and LON compression by the modified bone structure of the cranial junction and/or muscles and ligaments of the nuchae. The spreading of pain to the V1-trigeminal region would be related to the overlap of neurons and synapses of the C2 and C3 roots with trigeminal neurons observed in the trigeminal spinal complex.

As we could observe in this patient, several authors have shown improvement in headaches after surgery with a consequent decrease in swelling. Nevertheless, when GH levels don’t normalize after surgery, the headache usually persists, suggesting an association between headache and GH, which could be explained by the maintenance of the edema. In summary, this case emphasizes that unilateral headaches in patients with acromegaly may be related to nerve entrapment by soft tissue edema in the nuchal region. Some authors have described the headache in acromegalic patients as SUNCT, Horton, or hemicrania continua. We would like to emphasize that our patient does not have the characteristics of these rare diseases, whose prevalences are respectively 0.06%, 0.1%, and 1.8% [13-15], and as far as we are concerned, there is no association between acromegaly and neuralgic headaches.

## Conclusions

This paper describes a patient with frequent episodes of cervicogenic-like headaches during the four years before the diagnosis of acromegaly. Further studies are necessary to analyze the C2 and C3 compression caused by edema in nuchal muscles or alterations in the craniocervical junctions causing transitory peripheral neuropathy in acromegalic patients.
